# Development and validation of the Manipal Malocclusion Questionnaire (MMQ): a mixed-methods study among Indian adolescents

**DOI:** 10.1186/s12903-025-06603-0

**Published:** 2025-08-11

**Authors:** Saloni Agarwal, Supriya Nambiar, Anwesha Mishra, Rajath Rao

**Affiliations:** 1 Department of Orthodontics and Dentofacial Orthopaedics, Manipal College of Dental Sciences Mangalore, Manipal Academy of Higher Education, Manipal, Karnataka 576104 India; 2 Department of Community Medicine, Kasturba Medical College, Mangalore, Manipal Academy of Higher Education, Manipal, Karnataka 576104 India

**Keywords:** Malocclusion, Quality of life, Adolescents, Tool, Measurement

## Abstract

**Background & objectives:**

To develop and validate a structured, reliable malocclusion-related quality of life-specific questionnaire and measure its reliability in adolescents of Indian population.

**Materials & methods:**

A mixed methods approach with the sequential exploratory design was followed. Focus group discussions were conducted with orthodontists attached to teaching hospitals, patients aged (12-18 years) and their guardians. The interviews were recorded, transcribed, and analyzed using framework analysis. Several themes and sub-themes identified were used to identify items for the new tool, which was tested on 282 adolescents for its validity and reliability. Normative need for treatment was measured using the Index of Orthodontic Treatment Need (IOTN) and Handicapping Malocclusion Assessment Record (HMAR) index.

**Results:**

Five themes generated from qualitative data collection were self-perception, social interaction, difficult experiences with malocclusion, Missed opportunities, Patient awareness and expectations. Cross-sectional testing of the new 51-item-MMQ was found to be reliable (Cronbach’s α of 0.899) and construct validation using IOTN–DHC (*p* < 0.05) and DAI (*p* < 0.01) was statistically significant.

**Conclusion:**

The new tool named Manipal Malocclusion Questionnaire (MMQ) contains 51 items arranged in 5 domains which along with the socioeconomic scale according to Kuppuswamy socio-economic status scale 2021 was found to be reliable and valid for the tested Indian population. This tool can be of clinical relevance to understand patient expectations, motivation and outcomes in orthodontics and also for better quality assurance.

## Manuscript

Quality of life (QOL) refers to ‘an individual’s sense of well-being which is influenced by their satisfaction or dissatisfaction with various aspects of life that matter to them. Since health plays a crucial role in shaping one’s quality of life, the effect of health and disease on well-being is referred to as health-related quality of life (HRQL) [1]. This is essential in those treatments that are recognized as ‘cosmetic’ or ‘elective’. Health-related quality of life has many aspects of an individual’s life that are not accessible to the doctor and, therefore, the patient is the best person to judge their own HRQL.The decision as to whether malocclusion and orthodontic treatment fit into the classic concept of health and disease is a difficult one. One common reason to undergo orthodontic treatment for patients is improvement in aesthetics and psychosocial well-being. Given that malocclusion primarily affects aesthetics and psychosocial well-being rather than cause physical symptoms, there is a growing need to assess the condition’s impact through patient-centered quality of life tools rather than clinical indices alone. Research in orthodontic treatment has leaned on traditional indices and measurements (for example, PAR scores or cephalometric measures before and after treatment) or measures of morbidity (for example, root resorption following treatment) [2, 3]. These clinical indicators are of importance but require addition of HRQL measures because the HRQL demand, need not correlate with objective findings and patients ratings of demands may not be same as those of clinicians. For these reasons self-reported HRQL instruments should be used to ensure that patient’s own views/feelings are measured [4]. With the fact that, most often orthodontic patients are children/young adolescents, there may be some hindrances to the use of existing HRQL measurement tools, as it may be lengthy, complex and contain items that appear irrelevant to the respondent. According to previous literature, there have been several attempts to explore the multifaceted impact of dentofacial deformities and malocclusion on oral health–related quality of life (OHRQoL). Cunningham et al. [4] identified four core themes: emotional well-being, social interactions, functional limitations, and aesthetic concerns. Similarly, O’Brien et al. [2] focused on the social aspects of deformity**,** including facial aesthetics, functional issues**,** and awareness of facial differences. Choi et al. [3] and Feu et al. [5] based their studies on the OHIP-14 questionnaire, identifying seven key domains: functional limitation, physical pain, psychological discomfort, physical disability, psychological disability, social disability, and handicap**,** reflecting a more comprehensive interpretation of OHRQoL impacts and reinforcing the relevance of these dimensions in understanding the quality of life outcomes associated with orthodontic treatment need and treatment-seeking behaviour. Broder et al. [6] in 2007 developed the Child Oral Health Impact Profile to assess the oro-facial well-being in school children containing 34 items across 5 domains: oral health, functional well-being, social/emotional well-being, school environment and self-image. It emphasized on general health-related quality of life with emphasis on pathological concerns or functional concerns associated with pathology but did not include malocclusion specific concerns like aesthetics or psychosocial impacts of malocclusion. A study by Kok et al. [7] compared the Aesthetic Component of IOTN with Child Perceptions Questionnaire to assess orthodontic treatment needs and concern. This study found low correlation between examiner-rated IOTN scores and self-perceived quality of life. This highlights a limitation in using CPQ or IOTN AC alone, as neither fully captures both clinical severity and psychosocial impact—important for orthodontic treatment decisions. Another cross-sectional study conducted by O’ Brien et al. [2] evaluated the validity and reliability of the Child Perceptions Questionnaire (CPQ) as a measure of oral health-related quality of life (OHRQoL) in adolescents with malocclusion which highlighted the need to develop a condition-specific form specifically for prospective orthodontic patients. A study by Patel et al. [8] focused on developing a Malocclusion Impact Questionnaire (MIQ) to assess the oral health-related quality of life (OHRQoL) in adolescents with malocclusion specific to the population of United Kingdom. Most of these scales aren’t suitable to orthodontic patients as they focus on disease, pain and discomfort whereas orthodontics often addresses malocclusion based on societal norms rather than disease. And none of these scales were specific to the Indian population. For these reasons, use of malocclusion-specific measures with a small number of relevant items should be undertaken [9]. Thus, the authors aimed to develop a valid and reliable structured tool for determining malocclusion related quality of life in adolescents.

## Objectives

- To seek perception of orthodontists in and around Mangalore on deleterious effects of malocclusion on a patients’ quality of life and on factors that determine the need for orthodontic treatment
- To gain multiple perspectives of adolescents regarding impact of malocclusion on functional, aesthetic and social aspects of their everyday life.
- To measure perceived need and wish of orthodontic treatment with normative indices including Handicapping malocclusion assessment record and Index of orthodontic treatment need
- To incorporate these views into a new malocclusion specific questionnaire and to measure its reliability and validity in adolescents of Indian population

## Materials and methods

### Participants, eligibility criteria and study setting

**Fig. 1:**
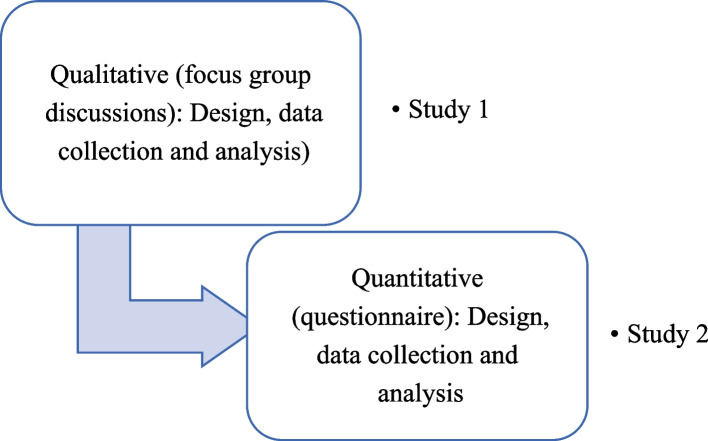
Summary of the mixed-methods approach employed

The present study used a sequential exploratory mixed methods research design (Fig. 1) approach to address the research aims. It comprised of a qualitative, and a quantitative component carried out from October 2019—January 2022 after obtaining approval from the Institutional Ethical Committee (Protocol Reference Number:19102). In the current study, the initial qualitative part comprised of three focus group discussions with orthodontists and two focus group discussions with patients and their respective guardians, in a non-clinical area setting with one moderator and one video recording personnel present. Orthodontists were purposely recruited on the basis of their association with a dental college with designated orthodontic department. Initially, 6 orthodontists were recruited for the first pilot focus group discussion based on the themes identified from the existing literature evidence. After the initial focus group discussion had been analysed, orthodontists were purposely recruited to inform and broaden some of the emerging themes and ideas from the earlier interviews. In total, 17 orthodontists took part in the study. All orthodontists were approached in the college setting by principal investigator and after consent was given an information sheet explaining each aspect of the research. Recruitment of participants ended when saturation occurred – that is, successive interviews were offering no new insights or challenges to the developing ideas and themes.

**Fig. 2:**
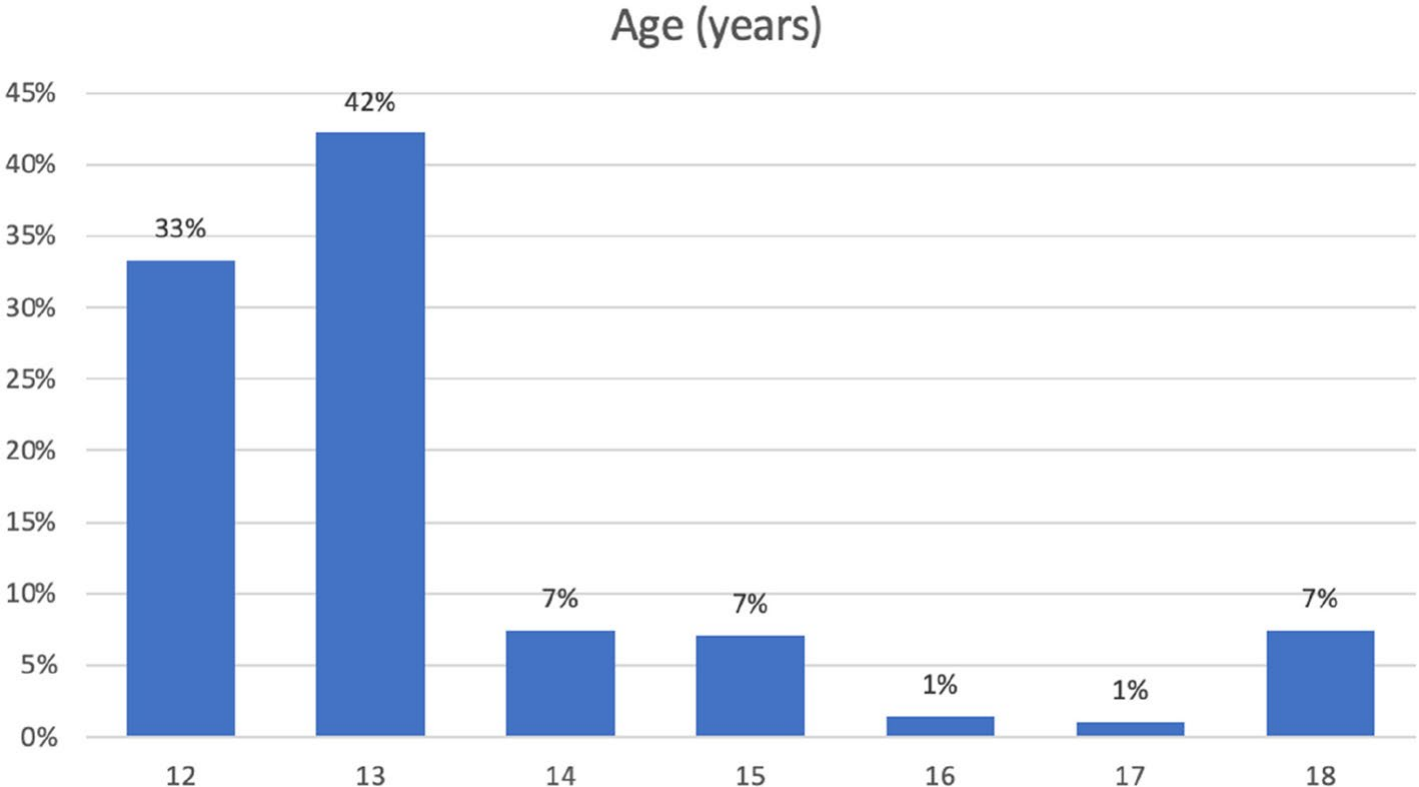
Bar graph showing the age distribution of the respondents (patients)

**Fig. 3:**
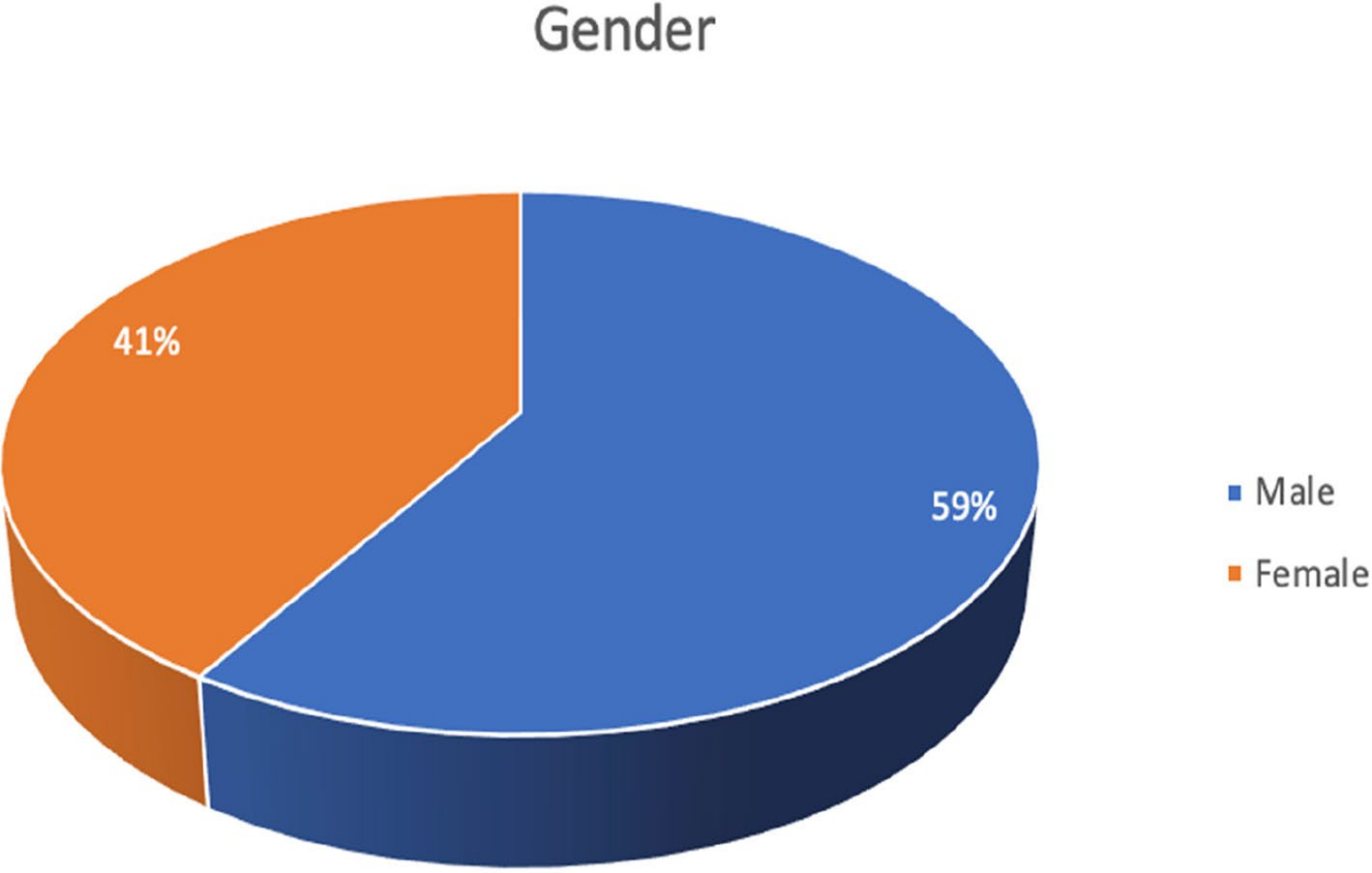
Pie chart showing the gender distribution of the respondents (patients)

**Fig. 4:**
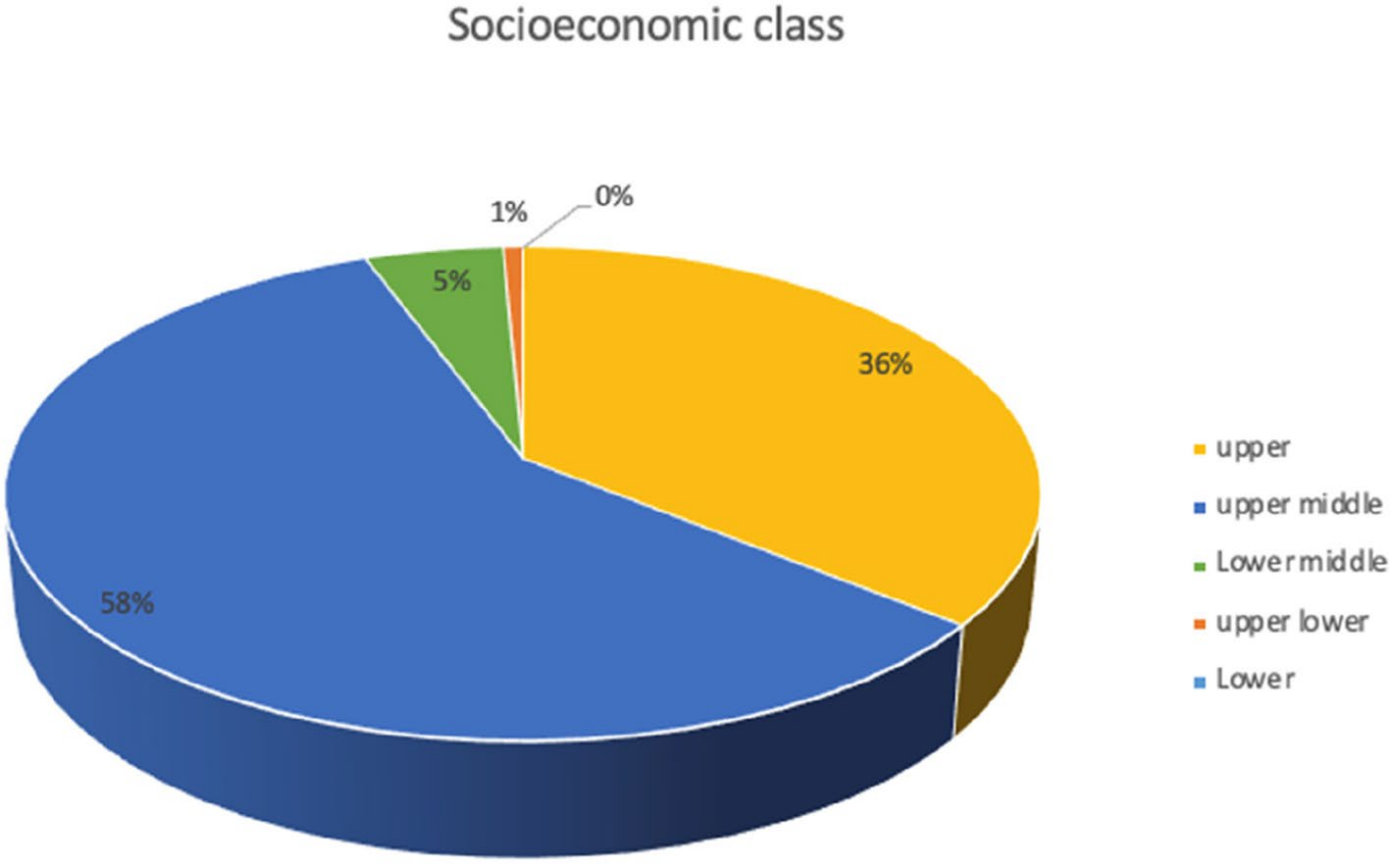
Pie chart showing the socioeconomic class distribution of the respondents (patients) according to Kuppuswamy socio-economic status scale 2021

**Fig. 5:**
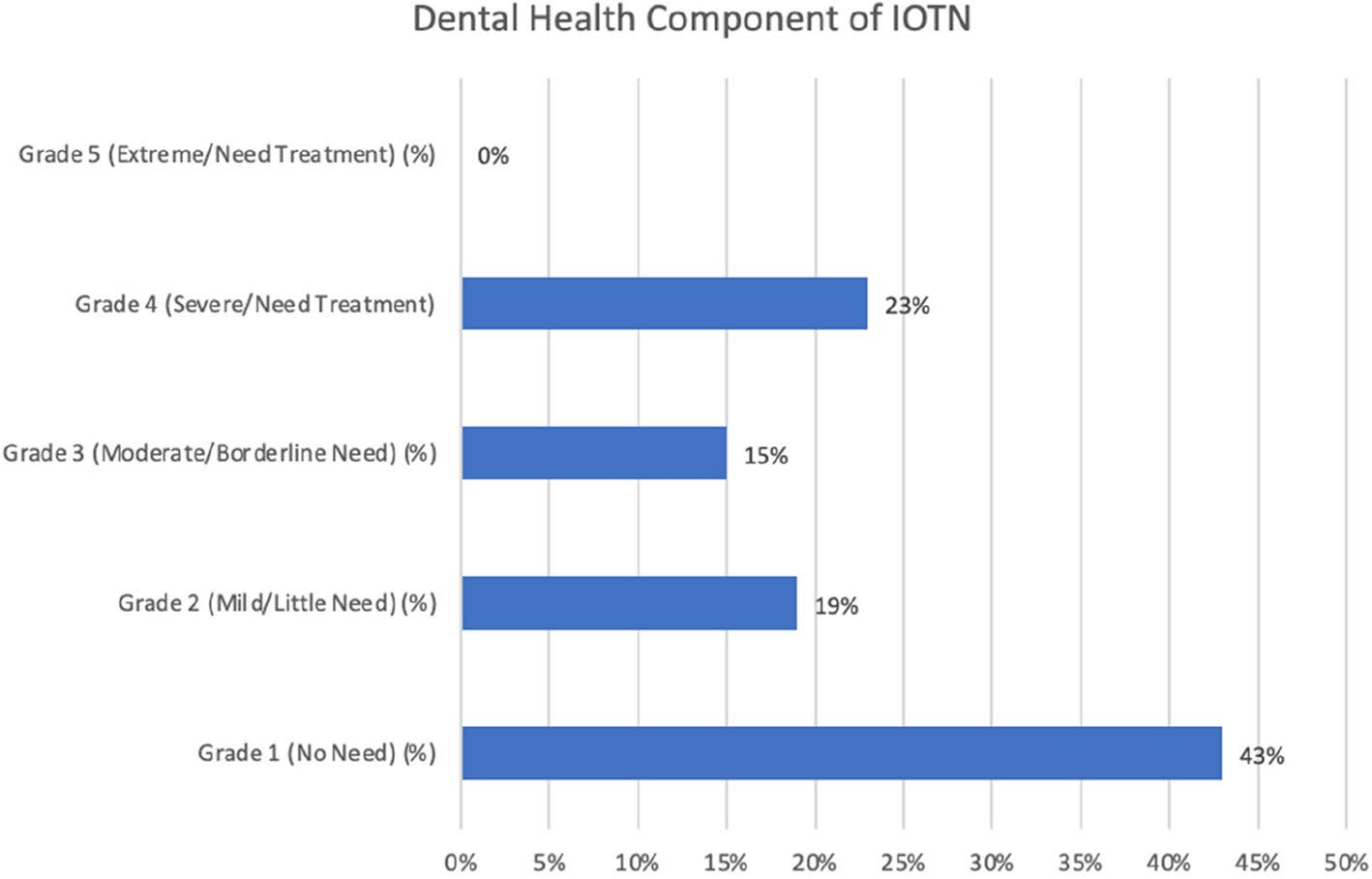
Bar graph showing distribution of different grades of Dental health component of IOTN

The focus group discussion for the patients aged 12 to 18 years was conducted in a non-clinical set up. The parents signed the consent form, while the children signed an assent form. In total 9 adolescents and 5 guardians were chosen by purposive sampling and with consent, to participate in the study. All the focus group discussions were audio and video recorded after obtaining the participants'consent and were carried out in person. For all focus group discussions an initial guide was followed by the moderator with the introduction of the study, laying down of ground rules, opening questions, deep core questions followed by less sensitive closing questions. As the study progressed, the guide was adapted further to explore emergent topics. The focus group discussions ranged from 40 min to one hour in duration and were carried out till saturation was reached. No new data emerged after which the focus group discussions were ceased. All the focus group discussions were transcribed by the investigator using MS Word and were deidentified and numbered for confidentiality purposes. The transcripts were coded using MAXQDA (MAXQDA Analytics Pro 2022; Release 22.0.1, 2021 VERBI GmbH, Berlin, Germany) [10] software, by the investigator. In order to reduce bias, 20% of transcripts were independently coded by a second orthodontic researcher, and discrepancies were discussed until consensus was reached. Thematic analysis was done using the code-based analysis approach. The 5 themes were generated from the codes using an inductive and deductive approach which included self-perception, social perception, difficult experiences with malocclusion, patient motivation, patient awareness and expectations of orthodontic treatment. These themes were used as domains to develop a questionnaire. Item generation was done from existing literature [1, 4, 6] and items identified in qualitative data collection. A matrix was created linking each item to supporting qualitative quotes and literature references. For example, Item 8 (‘Are you happy with your smile?’) was derived from direct quotes in Theme 1 (Self-perception). The questionnaire consisted of 51 items divided into five domains. The responses for most items of the questionnaire were developed using Likert scaling of definitely yes, yes, maybe, no, not at all. The initial part of the questionnaire included the Modified Kuppuswamy Scale for the year 2021 [11] for determining the socioeconomic class of the individual. The questionnaire was content validated by experts. To assess reliability, the questionnaire was administered to a sample of 282 school-going children aged 12 to 18 years (Fig. 2) of either gender (Fig. 3) and varying socioeconomic statuses (Fig. 4, Table 1), excluding children who had previously undergone any orthodontic treatment or had a complex medical history or learning disability that would impair understanding of the questionnaire. The normative malocclusion features and treatment need were recorded by the principal investigator using 2 indices: Index of Orthodontic Treatment Need (IOTN) comprising of Dental Health Component (DHC-IOTN) and Aesthetic Component (AC-IOTN) And Handicapping Malocclusion Assessment Record (HMAR) index [12]. The Aesthetic Component of IOTN (AC-IOTN) was filled out by the respondents. All examinations were performed under natural light setting in a non-clinical area using a sterilised mouth mirror by the investigator (Fig. 5).

**Table 1:** Frequency table showing socioeconomic class distribution of the respondents according to Kuppuswamy socio-economic status scale 2021

	Frequency	Percent	Percent (valid)	Percent (cum.)
Upper (I)	101	35.80	35.80	35.80
Upper middle (II)	165	58.50	58.50	94.30
Lower middle (III)	14	5.00	5.00	99.30
Upper Lower (IV)	2	0.70	0.70	100.00
TOTAL	282	100.00		

## Statistical analysis

Inductive thematic approach was performed for data collection. The qualitative data was analysed using MAXQDA (MAXQDA Analytics Pro 2022; Release 22.0.1, 2021 VERBI GmbH, Berlin, Germany) software. Initially, data familiarization was done followed by generation of initial codes, theme search, theme review, final themes and sub themes naming. Flesch-Kincaid Grade Level test (Kappa statistics) was done to determine the reliability of the questionnaire. For the quantitative data, for the comparison between scores obtained in the tool with the indices, Pearson correlation was used. Responses were subjected to factor analysis using squared multiple correlations as prior communality estimates. The principal component method of factor extraction and varimax method of factor rotation was used. For content validity, around 5 subject experts were consulted for their opinion regarding relevance, clarity, and ambiguity. The Content Validity Index (CVI) of the entire Scale (CVI-S) was 0.79, indicating that the items are generally relevant. Exploratory factor analysis: Sample adequacy measured by: KMO Value: 0.07, Multicollinearity as measured by Bartlett's test of sphericity: *p* < 0.001 (multicollinearity exists), model chi-squared test: *p* < 0.001.

## Results

### Theme 1: self-perception

In this study thirty-one participants were interviewed (17 orthodontists. 9 adolescents with ages ranging from 12 to 18 years and 5 parents). The themes identified from the interviews and the results are presented below according to these major concerns: Self perception, Social perception, Difficult experiences with malocclusion, patient motivation and patient awareness and expectations.

In this theme, it was observed that most participants had a negative self-perception (quote 1 and 2). Orthodontists reported of positive self-perception from patients post orthodontic treatment (quote 3 and 4).

1. *My face is not in good shape (Patient 4, FGD-1, Male, age 12 years)*
2. *“I don’t like my teeth to be this front.” (Patient 2, FGD-2, Male, 12 years)*
3. *I've seen a drastic improvement in their confidence much much more than any dental or skeletal parameters (Orthodontist 3, FGD-1, Male, 36 years)*
4. *self-confidence has improved because of their good surgical outcome (Orthodontist 6, FGD 1, Male, 37 years)*

### Theme 2: social perception

It was observed that patients were very insecure about how people perceived them especially with regards to their smile. This restricted their social interaction and made them timid.

1. *“So, when I’m taking photos, I got conscious and got insecure because of that. So, I did not like taking pictures, cause it kind of looks weird and I want to fix that first.” (Patient 4, FGD-2, Female, 17 years)*
2. *“Presently everyone wants to take selfies. They are overly concerned by themselves all the time. And they over analyse themselves for having smaller issues because they want to look good on social media.” (Orthodontist 5, FGD-3, Female, 36 years)*
3. *“Patients with severe skeletal problems are timid. Conscious about the smile. Not very socially active. In such patients, once the treatment is over, you see a great change in their confidence and social conduct” (Orthodontist 6, FGD-3, Male 39 years)*
4. *“I: Do you restrict yourself from taking pictures and all? Patient 4: Actually, yes, I do. Kind of.” (Patient 4, FGD 2, Female, age 17 years)*

### Theme 3: difficult experiences with malocclusion

Orthodontists identified more with the health implications of malocclusion, where as parents were more concerned about the appearance of teeth and how it impacted their children in their day to day activities. The adolescents were very vocal on how their malocclusion affected them at school, the bullying they experienced from peers and also few of them spoke on difficulties during biting and trauma.

#### Expressed by orthodontists

1. *“A lot of open bite cases they complain of not being able to bite from the front, chew food from the front” (Orthodontist 5, FGD-1, Male, age 29 years)*
2. *“Crowding leads to food lodgement, the tooth is going to get decay and then it is going to go for root canal treatment.” (Orthodontist 5, FGD-3, Female, 36 years)*
3. *“Crowding they usually have halitosis.” (Orthodontist 2, FGD-2, Female, 30 years)*
4. *“I think issues like snoring are really bothering the patient’s personal life. Partners they are uncomfortable.. Such issues are really uncomfortable.” (Orthodontist 3, FGD2, Male, 37 years)*

#### Expressed by patients

1. *“My bite is actually not proper; I have an open bite and one side of my jaw is longer than the other side and I’m having a lot of pain because of that while biting. I didn’t bother that much about my smile. It hurts really badly.*
  I: *Did it affect your sleep? Yes, definitely very much* *” (Patient 4, FGD-2, Female, age 17 years)*
2. *“* *My teeth are in front so I can’t close my lips.* *It’s difficult to close lips* *” (Patient 2, FGD-2, Male, age 12 years)*
3. *“* *One teeth broke, back in my village when I slipped and fell down the floor* *” (Patient 4, FGD-1, Male, age 12 years)*
4. *“* *So that when I brush, I can brush comfortably* *” (Patient 2, FGD 1, Male, age 13 years)*

#### Expressed by parents

1. *“If I see his teeth, one tooth is inside one tooth is outside, the shape is not correct “*
2. *“So, when front teeth came, he used to keep his mouth in a different way, not in a good way. He would keep his mouth open and sleep.” (Patient 2 and 3’s father, FGD2, male, 42 years)*

Adolescents provided several instances of bullying and mockery and calling of names which they experienced irrespective of whether they were from a rural or urban area. Bullying experienced by the children due to their malocclusion made them embarrassed about themselves which they expressed to their parents as concerns. The orthodontists were able to perceive the trauma children experienced mostly through the parents and also in some instances through the children themselves.

1. *“Actually, in my village, they say “todi pallu”, the girl who has her teeth in front, they tease me. I remember that and told my mom to make my teeth proper, so my mother got me here for treatment (Patient 3, FGD 2, Female, age 12 years)*
2. *“When I went to school, the other kids started teasing me that my teeth are in front. I felt my teeth are in front so I can’t close my lips. Yeah, it was bad, but I never cried, if I did, they will make even more fun of me (Patient 2, FGD 2, Male, age 12 years)*
3. *“I mean now it’s like we are older right. So, I feel like now if I was in 5th standard or something, I would have probably gotten made fun of but now we are like mature.” (Patient 4, FGD 2, Female, 17 years)*
4. *“My father stays in a village, so when I go to my village for the summer holidays, my village friends always tease me that why are you showing your rabbit teeth, so I feel bad about it” (Patient 3, FGD-2, Female, 12 years)*
5. *“My friends say that when I talk, my teeth look like monster’s teeth” (Patient 2, FGD 1, Male, age 13)*
6. *“When he grows up, no one should mock or comment. No one does now, but even if anyone does, he wouldn’t understand that.” (Patient 4’s mother, FGD-1, 40 years, Female)*
7. *“When I used to make fun of him, he used to tell me to take him to the dentist and get my treatment done” (Patient 2 and 3’s father, FGD-2, Male, 42 years)*
8. *“Emotional state of the child is usually told by parents, that he’s getting bullied, or he is sad, not from the patient himself or herself, unless the patient is 15-year-old or above. (Orthodontist 6, FGD 3, 39 years, Male)*
9. *“So, a lot of these kids suffer silently you know I'm sure inside they're hurting about it, and tell that such things have happened to them” (Orthodontist 3, FGD 1, Male, 36 years)*

### Theme 4: patient motivation for treatment

According to the orthodontists, motivation for orthodontic treatment, mostly from parents who were worried for their children. social media and peer influence also played an important role in treatment decisions. Adolescents attributed their motivation to how their parents and near and dear ones perceived them. Stigma, rural -urban divide, socioeconomic status, treatment duration and complexity were the factors identified by orthodontists that played an important role in patient motivation.

1. *“A lot of paediatric cases come in, where parents are more worried for their kids even when they themselves have malocclusion. They couldn’t get it treated but they want their kids to be treated.” (Orthodontist 5, FGD 3, 36 Female)*
2. *“A lot of times it's not the patient who has a problem, it is the people around them. It will give an idea of where they got motivated initially.” (Orthodontist 6, FGD 1, Male, 37 years)*
3. *“Another thing is that if one sibling has already had treatment, somehow the next also think that it's a rite of passage and parent bring them for the next appointment so that everybody can get the treatment done together.” (Orthodontist 1, FGD 2, male, 54 years)*
4. *“It’s social media-driven most of the time in these days, they want to look better, they want better pictures.” (Orthodontist 1, FGD 3, male, 32 years)*
5. *“Sometimes cousins or peers get the treatment done and they get motivated.” (Orthodontist 2, FGD 3, Male, 28)*
6. *“Because my teeth are In front, my mother wanted me to get the treatment done” (Patient 3, FGD 1, Female, age 14)*
7. *“Because you conducted a camp at our school that’s why.” (Patient 1, FGD-1, 15, Male)*
8. *We came here cause the treatment charges are low” (Patient 2’s mother, FGD-1, Female, 40 years)*
9. *“We came to get his milk teeth removed, they said that his permanent teeth are behind, and he will have to get braces done. So, we got referred here” (Patient 2’s mother, FGD-1, Female, 40 years)*
10. *“We have reimbursement from an insurance company, s and personally a lot of our employees get their treatment done here and have said it is nice here and recommended coming here”. (Patient 4’s mother, FGD-1, Female, 40 years)*
11. *“When my mother was getting her braces done, the doctor had told her that your son should be of an age at least 14 to get my braces” (Patient 1, FGD-1, Male, 15 years)*
12. *“I wanted to come because my brother got treated here (Patient 2, FGD 1, Male, 13 years)*

### Theme 5: patient awareness and expectations regarding orthodontic treatment

There were a lot of concerns from adolescents regarding pain and difficulty during treatment period. Parents were unaware of the positive outcomes outcomes that can result from malocclusion correction. Orthodontists stated that there has been an increased awareness with the advent of social media but still patients are more concerned of their aesthetics more than any other aspects of malocclusion.

1. “*Will any weakening happen in old age? When we disturb the location, some gap will be there, some bone loss or something will happen? “ (Patient 2 and 3’s father, FGD2, Male, 42 years)*
2. *“During treatment, I might not be able to eat hard foods, my brother is not able to.” (Patient 2, FGD 1, Male, 13 years)*
3. *He might have pain and have difficulty in eating” (Patient 4’s mother, FGD 1, Female, 40 years)*
4. *“I didn’t know that I needed treatment” (Patient 5, FGD 1, Male, 15 years)*
5. *“While brushing it might be difficult to brush” (Patient 3, FGD 1, Female, 14 years)*
6. *“Before 90 s patients would live with it. They used to accept these factors but right now because of education maybe digital era internet and other things awareness is increased”. (Orthodontist 3, FGD-2, Male, 37 years)*
7. *“They will be class 3 but just want their teeth to go back, but when we explain that by just doing this your profile won't improve at the end, what they want is an aesthetic makeover which won't happen”(Orthodontist 6, FGD 1, Male, 37 years)*
8. *“In rural areas, there is stigma related to extraction. It will lead to head problem or eye problem. So, when patients come with chief complaint of proclination and you tell them you have to extract four teeth then, they do not agree for it. It’s difficult to get rid of the stigma.” (Orthodontist 3, FGD 3, Male, 30 years)*
9. *“They complain that the uppers are proclined but it's usually the mandible that is backward placed. When you explain to them, usually around 70% of the patient do agree to get the treatment “(Orthodontist 7, FGD 3, Male, 31 years)*
10. *“It’s difficult to convince them regarding period of the treatment. Treatment time is an important factor which usually patients don’t accept.” (Orthodontist 7, FGD 3, Male, 31 years)*
11. *“Patient awareness is very good in urban areas. A lot of clinics, lot of camps are happening. If you go to a rural place, that awareness is not there. Another factor is cost” (Orthodontist 6, FGD-1, Male, 37 years)*
12. *“Patients underplay the severity of the malocclusion. patients generally tend to consider it less severe than what it actually is.” (Orthodontist 4, FGD-1, Male, 43 years)*
13. *“Sometimes patients want only simple corrections to be done,however complex the malocclusion is.” (Orthodontist 5, FGD-3, Female, 36 years)*
14. *“Surgery as a treatment plan is generally not accepted. They feel it’s something major and life threatening.” (Orthodontist 5, FGD-3, Female, 36 years)*
15. *“Parents are less interested in growth modulation thinking later on when fixed orthodontic treatment is anyway needed. They fear the child may have compliance issues.” (Orthodontist 6, FGD-3, Male, 39 years,)*

The items were developed using the qualitative data and the existing literature. The themes were categorised as domains in the questionnaire.

Descriptive statistical results of age, domain scores (Self-perception score, Social-perception score, Difficult experiences with malocclusion score, missed opportunities score, Patient motivation score) and total score of the questionnaire filled by the 282 respondents is shown in (Table 2).

**Table 2:** Descriptive statistical results of age, domain scores (Self-perception score, Social-perception score, Difficult experiences with malocclusion score, missed opportunities score, Patient motivation score) and total score of the questionnaire filled by the respondents

	N	Mean	Std. dev. (pop.)	Minimum	Maximum
Age	282	13. 34	1. 657	12	18
Self-perception score	282	33. 18	5. 030	22	49
Social-perception score	282	30. 95	6. 409	15	49
Difficult experiences with malocclusion score	282	48. 70	8. 463	28	75
Missed opportunities score	282	8. 37	2. 571	4	16
Patient motivation score	282	2. 52	1. 117	1	5
Aesthetic component of IOTN	282	2.62	1. 984	1	9
Dental health component of IOTN	282	2.19	1. 216	1	4
HMAR	282	13.99	10.119	1	36

**Fig. 6:**
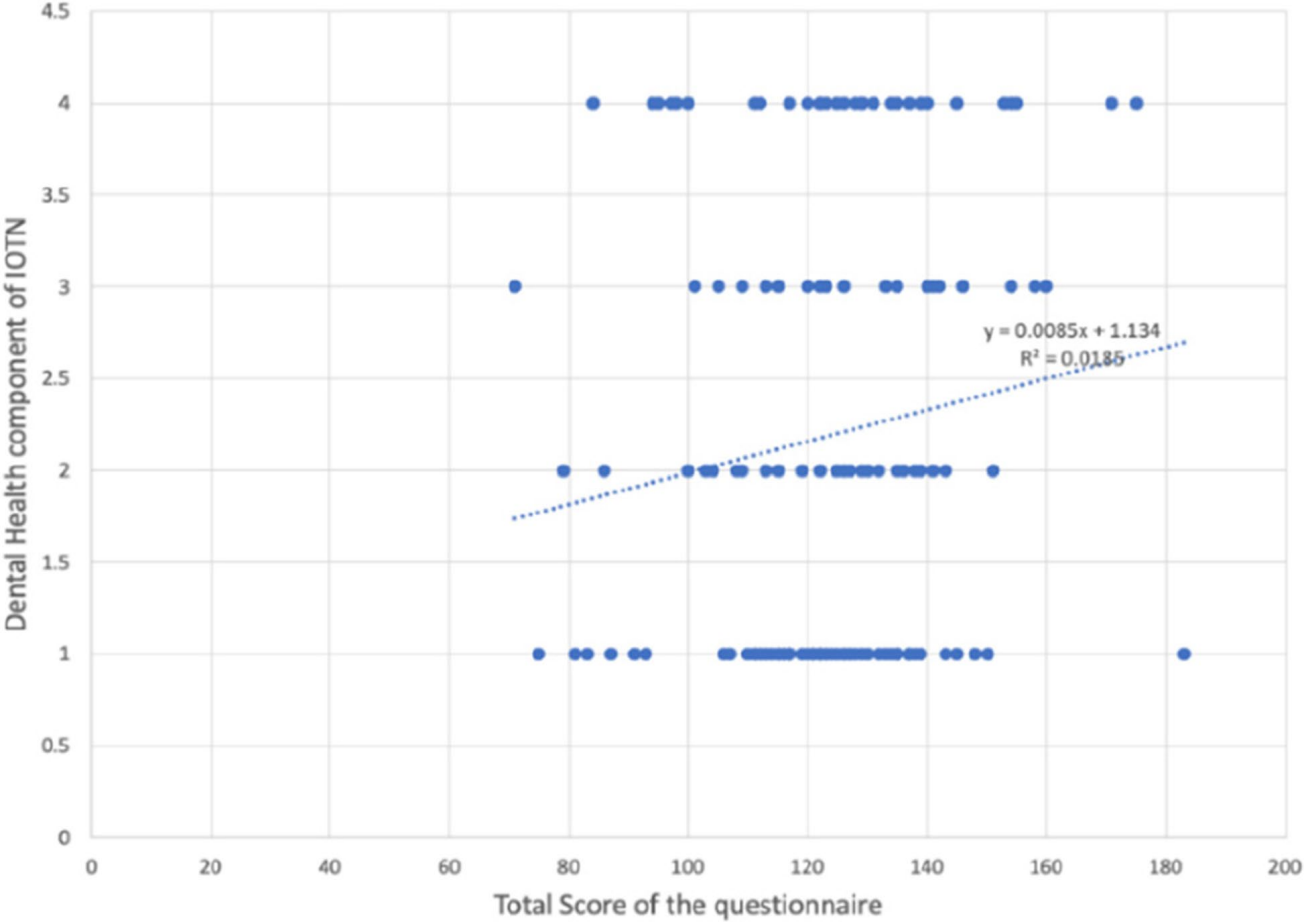
Scatter plots showing relation between Total score of the questionnaire with Aesthetic component of IOTN, Dental Health component of IOTN and HMAR index

**Fig. 7:**
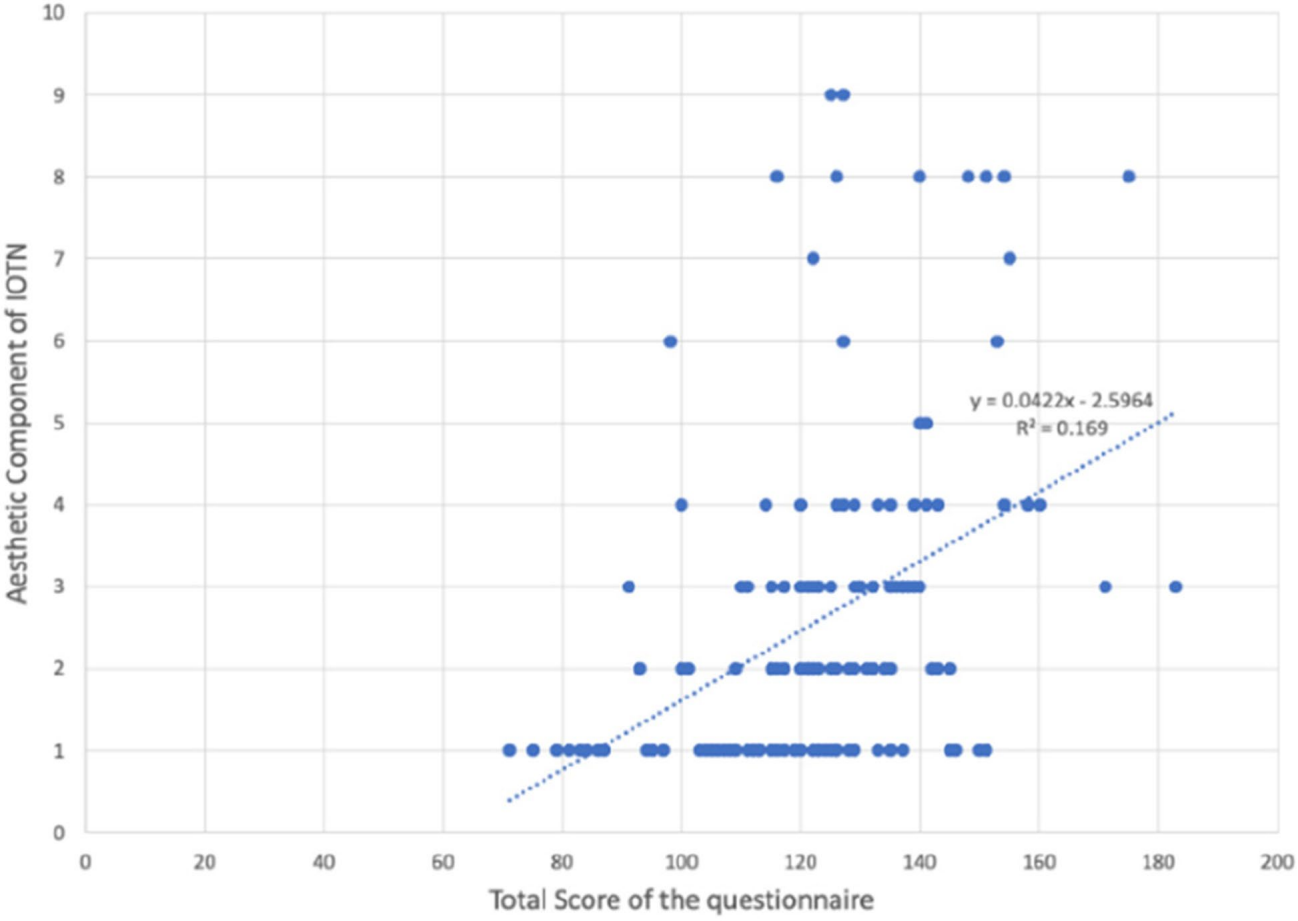
Scatter plots showing relation between Total score of the questionnaire with Aesthetic component of IOTN, Dental Health component of IOTN and HMAR index

**Fig. 8:**
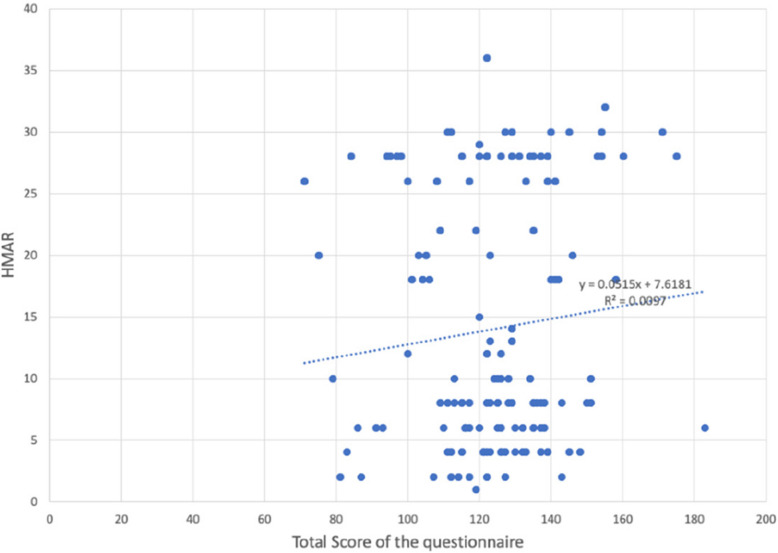
Scatter plots showing relation between Total score of the questionnaire with Aesthetic component of IOTN, Dental Health component of IOTN and HMAR index

Reliability statistics of the 51items (Q1-Q51) of the questionnaire an overall Cronbach’s alpha of 0.899 as shown in (Table 3). The distribution of different grades of Dental health component of IOTN among the 282 particpants is shown in (Table 4)  (Figs. 6, 7, 8 and Table 5).

**Table 3:** Reliability statistics of the 51items (Q1-Q51) of the questionnaire with an overall Cronbach’s alpha of 0.899

Item-Total Statistics				
	Scale Mean if Item Deleted	Scale Variance if Item Deleted	Corrected Item-Total Correlation	Cronbach's Alpha if Item Deleted
1. Do you look at your teeth often in the mirror?	120.01	369.879	.105	.900
2. Do you look at your face often in the mirror?	119.69	368.799	.153	.899
3. Do you think your teeth are projecting out?	121.11	356.466	.451	.896
4. Do you wish your teeth looked better?	120.22	356.201	.388	.897
5. Do you think your teeth are crooked?	121.04	355.408	.445	.896
6. Do you have gaps in between your teeth?	121.10	360.303	.301	.898
7. Do you enlarge your pictures and think that your teeth could look better?	121.05	355.421	.397	.897
8. Are you happy with your smile?	121.43	355.107	.528	.895
9. Do you like the way your face looks from the side view?	121.23	360.522	.406	.897
10. Do you think you look better when your lips are closed or when your teeth are 2 t showing?	120.46	365.687	.182	.899
11. Are you self-conscious about your face?	120.37	363.878	.229	.899
12. Have you ever been told that you should get braces?	120.92	353.453	.414	.897
13. Do you want to get braces done only because you have seen other people get them?	121.68	364.289	.275	.898
14. Do you shy away from making new friends or talking to new people?	121.30	353.394	.465	.896
15. Do you shy away from taking photographs or selfies?	121.18	355.083	.405	.897
16. Do you avoid talking to the other gender?	121.50	364.578	.242	.899
17. Do you cover your mouth when you laugh?	121.06	354.295	.480	.896
18. Do you cover your mouth when you eat?	120.87	360.510	.269	.899
19. Do you avoid eating in public?	121.21	354.887	.461	.896
20. Have you been singled out in a group because of how you look?	121.84	362.827	.385	.897
21. Do you think, or have you been told that a lot of your gums are visible when you smile?	121.73	364.625	.305	.898
22. Do you stare at other people's teeth?	121.68	362.695	.302	.898
23. Do you envy the nice teeth of other people?	121.05	356.247	.403	.897
24. Do you avoid speaking out loud or reading in class?	121.32	352.745	.499	.895
25. Do you or have you been told that you s2re at night?	121.72	362.111	.328	.898
26. Do you sleep well at night?	121.59	364.400	.281	.898
27. Do you have difficulty in saying certain words?	121.21	359.257	.379	.897
28. Do you find it hard to brush your teeth because of the way their position is?	121.70	357.434	.539	.896
29. Do your gums bleed while brushing?	121.32	362.695	.301	.898
30. Do you have pain in your jaw or face?	121.77	362.714	.403	.897
31. Is your pain the most, early in the morning when you wake up or during winters?	121.72	358.154	.521	.896
32. Do you find it hard to bite or chew certain foods?	121.46	358.513	.409	.897
33. Do you think or has anyone told you that your mouth smells bad?	121.25	357.682	.433	.896
34. Does food get stuck between your teeth?	120.53	358.456	.395	.897
35. Have you ever broken any front teeth because any fall or accident?	121.58	367.177	.130	.900
36. Do you or have you been told that you clench or grind your teeth at night?	121.64	369.029	.138	.899
37. Do you think your teeth do 2 t meet properly?	120.94	355.064	.459	.896
38. Do you find it hard to bite from the front teeth?	121.36	359.719	.334	.898
39. Do you hear grinding, popping or clicking sounds near your ear when you open and close your mouth?	121.33	365.573	.178	.900
40. Do you have difficulty opening your mouth?	121.99	366.754	.320	.898
41. Do you have difficulty closing your lips?	121.89	363.526	.408	.897
42. Do you or have you been told that you breathe from your mouth or to close your mouth?	121.46	358.271	.405	.897
43. Do you have difficulty in breathing through your 2se with your lips closed?	121.62	359.511	.417	.897
44. Do you get bullied at school for the way your teeth look?	122.04	363.215	.403	.897
45. Do people like your friends, relatives, family members call you names like rabbit because of your teeth?	121.67	362.031	.304	.898
46. Do comments about your teeth or face hurt you?	121.28	353.866	.453	.896
47. Have you restricted yourself from taking part in something because of the way your teeth or face looked?	121.60	353.422	.551	.895
48. Do you think you would perform better in school if your face or teeth looked better?	121.44	352.760	.525	.895
49. Have you restricted yourself from speaking in public because of difficulty in pro2uncing a few words?	121.54	354.769	.489	.896
50. Have you missed school because of pain in your jaw or face?	121.91	362.527	.375	.897
51. Have you ever wanted to get braces done for yourself?	121.20	354.750	.418	.896

**Table 4:** Frequency table showing distribution of different grades of Dental health component of IOTN

	Frequency	Percent	Percent (valid)	Percent (cum.)
Grade 1 (No Need)	120	42.60	42.60	61.70
Grade 2 (Mild/Little Need)	54	19.10	19.10	19.10
Grade 3 (Moderate/Borderline Need)	42	14.90	14.90	76.60
Grade 4 (Severe/Need Treatment)	66	23.40	23.40	100.00
TOTAL	282	100.00

**Table 5:** Pearson correlation coefficients (r^2^) for the relation of total score of the questionnaire with the Aesthetic component of IOTN Index, Dental health component of IOTN Index and the HMAR Index

	Socioeconomic class	Total Score of the questionnaire	Aesthetic component of IOTN Index	Dental health component of IOTN Index	HMAR Index
**Socioeconomic class**		0.092 (*p* = 0.1239)	0.151* (*p* = 0.0112)	−0.050 (*p* = 0.4036)	−0.073 (*p* = 0.2230)
**Total score of the questionnaire**	0.092 (*p* = 0.1239)		0.411** (*p* = 0.0000)	0.136* (*p* = 0.0225)	0.098 (*p* = 0.0990)
**Aesthetic component of IOTN Index**	0.151* (*p* = 0.0112)	0.411** (*p* = 0.0000)		0.136* (*p* = 0.0222)	0.129* (*p* = 0.0304)
**Dental health component of IOTN Index**	−0.050 (*p* = 0.4036)	0.136* (*p* = 0.0225)	0.136* (*p* = 0.0222)		0.860** (*p* = 0.0000)
**HMAR Index**	−0.073 (*p* = 0.2230)	0.098 (*p* = 0. 0990)	0.129* (*p* = 0. 0304)	0.860** (*p* = 0.0000)	

*Correlation is significant at the 0.05 level (2-tailed)

**Correlation is significant at the 0.01 level (2-tailed)

## Discussion

Is orthodontics compatible with medically necessary treatment? To derive an answer to this dilemma, we should consider the urgency of the medical diseases which are usually described in relation to the continuity of life (life and death issues) or quality of life. In most of the instances, dentistry and especially orthodontic treatment falls into the category of quality of life and this may have put the dental profession apart from the rest of the medical profession. However, pain moves dentistry into the limelight of medicine [9]. The debate of medically necessary orthodontic care evokes the question of whether malocclusion is malformation or malady.

Most of the orthodontic malocclusion severity indices, consider the occlusal features without considering this cephalometric measurements of the skeletal structure, facial soft tissue aesthetics or handicap or growth potential or psychological impact on the individual. The purpose of an OHRQoL instrument was not just to measure the presence and severity of disease symptoms, but also to show the impact of the illness and/or the intervention on that individual and, in some cases, to study unmet patient needs.

The current study aimed to develop a structured, malocclusion related quality of life specific questionnaire Manipal Malocclusion Questionnaire MMQ) for Assessing Malocclusion Related Quality of Life in Adolescents. A qualitative approach was carried to achieve this. Through structured interviews, it was possible to answer complex research aims such as perception of how malocclusion affects an individuals’ quality of life and questions more comprehensively, and to ‘offset’ the weaknesses of the two approaches while utilising their strengths. The research design of the current project most closely mirrored the ‘exploratory sequential design’. The sequential approach meant that the data collection and analysis of the initial qualitative study was completed prior to the design and conduct of the second, quantitative study.

For the qualitative data collection focus group discussions were conducted with seventeen orthodontists, nine patients and 5 guardians in total in a non-clinical setting. The name of the method defines its key characteristics, in that it involves a focus on specific issues, with a predetermined group of people, participating in an interactive discussion—thereby a focus group discussion. The method may be described as “an interactive discussion between six to eight pre-selected participants, led by a trained moderator and focussing on a specific set of issues so as to gain a broad range of views on the malocclusion related quality of life. In this study the context of a group discussion was thought to create greater impulsiveness in the contributions of participants because it replicated daily social interactions more than a traditional interview.

The function of nondirective interviewing was to swing the attention away from the dominance of an interviewer toward generating a discussion among participants. A framework approach to analysis was adopted for analysis of the qualitative data. This adhered to the following process of identification of themes wherein recurrent themes were identified and further developed.

Five main themes were identified from the focus group discussions which were, Self-perception, Social perception, Difficult experiences with malocclusion, Patient motivation and Patient awareness and expectations regarding orthodontic treatment. Upon development, the items were organised into themes. An initial thematic framework was constructed. The themes were categorised as domains in the questionnaire. Similar approach was followed by Cunningham et al. [1], Patel [8] and Javidi & Benson [13].

Concerns regarding appearance of the teeth was a consistent finding which were mainly related to the position of teeth particularly upper incisors. Patients reported of negative self-perception. These finding were consistent with findings of Phillips et al. [14], Claudino et al. [15]. Other studies have supported the finding that the clinical extent of the malocclusion does not appear to be related to self-concept [16] or self-esteem [17] in preadolescents and adolescents. Body image concerns among adolescents are strong, having influence in psychological and social adjustments, and educational success. Hence, age group selection was deemed important. It was decided that a new measure was needed as all the present generic measures of OHRQoL failed to capture the issues completely. Choi et al., 2015 [18] concluded that severe malocclusion is significantly associated with functional limitations, physical pain, and social disability in young adults. and that malocclusion is a key factor associated with poor quality of life caused by limited oral function, pain, and social disability in young adults. Al-Omari et al., 2014 [19] demonstrated a significant association between bullying because of dentofacial features and negative effects on oral health-related quality of life.

Henson et al. [20] demonstrated the differences in ratings between ideal and non-ideal smiles which were significant for perceptions of athletic performance, popularity, and leadership ability but not for academic performance. On average, ratings for the ideal smiles in perceived athletic, social, and leadership skills were about 10% higher than those given for images with nonideal smiles. They concluded orthodontic treatment resulting in improved smile esthetics can provide modest social benefits for adolescent patients.

In a systematic review Dimberg et al. [21] reviewed 4 studies with a high level of quality that showed anterior malocclusion had a negative impact on OHRQOL, and two studies with a moderate level of quality reported that increased orthodontic treatment need had a negative impact on OHRQOL. The scientific evidence was considered strong since 4 studies with high level of quality reported that malocclusions have negative effects on OHRQOL, predominantly in the dimensions of emotional and social wellbeing.

Another systematic review by Javidi et al. [13] concluded that orthodontic treatment during childhood or adolescence leads to moderate improvements in the emotional and social well-being dimensions of OHRQoL, although the evidence is of low and moderate quality. More high quality, longitudinal, prospective studies are needed. Kragt et al. [22] showed evidence for a clear inverse association of malocclusion with OHRQOL. They also showed that the strength of the association differed depending on the age of the children and their cultural environment.

The socioeconomic scale used was updated Modified Kuppuswamy SES” scale for the year 2021 [11, 23]. Concerns regarding appearance of the teeth was a consistent finding which were mainly related to the position of teeth particularly upper incisors. Body image concerns among adolescents are strong, having influence in psychological and social adjustments, and educational success. Hence, age group selection was deemed important. It was decided that a new measure was needed, as all the present generic measures of OHRQoL failed to capture the issues completely. The scale reliability refers to repeatability of the scale, and Cronbach’s α of 0.899 was found to be good as alpha of 0.7 or above is considered good for a new scale. Validity refers to measuring what a scale is intended and construct validation using IOTN–DHC (*p* < 0.05) and DAI (*p* < 0.01) was found to be statistically significant, ensuring the ability of the scale to distinguish those with treatment need and those without need. Although statistically significant, the weak Pearson correlation coefficient suggests construct validity might show a poor or mediocre fit indicating the need to modify the scale for a better fit.

## Conclusions

This new scale named Manipal Malocclusion Questionnaire (MMQ) contains 51 items arranged in 5 domains: self-perception, social interaction, difficult experiences with malocclusion, Missed opportunities, Patient awareness and expectations regarding orthodontic treatment with socioeconomic scale according to Kuppuswamy socioeconomic status scale 2021. The MMQ, a 51-item scale developed using a mixed-methods approach, demonstrated strong internal consistency and construct validity among Indian adolescents. Its use can inform treatment prioritization by integrating psychosocial burden with clinical need. Socioeconomic factors of patients seeking orthodontic treatment cannot be neglected in the light of this study. Low socioeconomic status magnifies the negative effects of malocclusion by limiting access to care, increasing psychosocial vulnerability, and reducing awareness or prioritization of orthodontic treatment. [17] Also, uptake of patients for subsidized treatment based on mere normative criteria is not enough; instead, the use of a psychometric tool reflecting socioeconomic status is recommended. This tool can be of clinical relevance to understand patient expectations, motivation and outcomes in orthodontics and also for better quality assurance and can also be used for improving the access of care for orthodontic patients.

### Limitations

Limitations of the study include few subjects with grade IV and absence of subjects with grade V of IOTN-DHC. Responses may be subject to social desirability bias, particularly in focus groups. Additionally, the tool was validated in a single geographic region; thus, generalizability to other Indian populations may be limited. Presence of other dental problems like dental caries and periodontal problems are potential confounders in the estimation of OHRQoL. Hence further validity and responsiveness testing of the scale is necessary.

## Data Availability

The datasets used and/or analysed during the current study are available from the corresponding author on reasonable request.
